# Machine learning enhanced acute heart failure phenotype prediction using natural language processing and random forest

**DOI:** 10.3389/frai.2025.1664627

**Published:** 2025-10-16

**Authors:** Pei-Hsuan Chang, Feng-Ching Liao, Yi-Ching Wu, Fang-Ju Sun, Yen-Yu Liu, Hung-I Yeh, Chung-Lieh Hung, Kun-Pin Wu

**Affiliations:** ^1^Institute of Biomedical Informatics, National Yang Ming Chiao Tung University, Taipei, Taiwan; ^2^Division of Cardiology, Department of Internal Medicine, MacKay Memorial Hospital, Taipei, Taiwan; ^3^Department of Medical Research, MacKay Memorial Hospital, Taipei, Taiwan; ^4^Department of Critical Care Medicine, MacKay Memorial Hospital, Taipei, Taiwan; ^5^Institute of Biomedical Sciences, MacKay Medical College, New Taipei City, Taiwan; ^6^Program of Interdisciplinary Medicine, National Yang Ming Chiao Tung University, Taipei, Taiwan

**Keywords:** heart failure, heart failure phenotypes, natural language processing, machine learning model, random forest

## Abstract

**Background:**

Heart failure (HF), with its distinct phenotypes, poses significant public health challenges. Early diagnosis of specific HF phenotypes is crucial for timely therapeutic intervention.

**Objectives:**

We employed random forests to predict acute HF (AHF) phenotypes (HFrEF, HFmrEF, and HFpEF) during admission, using structured and unstructured data types while blinded to left ventricular ejection fraction (LVEF) information.

**Methods:**

We investigated the predictive performance of integrated natural language processing (NLP) and machine learning (ML)-based models in AHF phenotype classification by random forests, leveraging clinical text and laboratory data from the MIMIC-III database. Feature selection for unstructured textual data and biochemical test data was performed using the LASSO method, with selected textual features converted into structured data using one-hot encoding. The areas under the ROC and PRC curves (AUROC and AUPRC) assessed overall performance.

**Results:**

Our final study cohort comprised 1,192 training datasets and 513 independent validating datasets with primary data types and LVEF information available. The overall model from the training dataset showed the best performance with combined datasets (accuracy: 0.70 ± 0.03, AUROC: 0.76 ± 0.02) compared to the textual or laboratory dataset alone, which was replicated in the independent validating dataset. Our model achieved optimal performance by selecting up to 100 combined features from both textual and laboratory data. Reducing features to 20 did not substantially attenuate the overall model performance until only 10 features were selected.

**Conclusion:**

Our study enhances HF phenotype classification and underscores the value of multifaceted data analysis in clinical informatics, enabling more personalized heart failure treatment. Early identification of AHF phenotypes may support timely, phenotype-specific management and inform treatment decisions.

## 1 Introduction

Heart failure (HF) is a global pandemic, affecting an estimated 64 million individuals and posing a significant public health challenge owing to its high prevalence (2.3% of adults), alarming mortality rates (approximately 10% annually), and substantial healthcare costs (exceeding $300 billion annually) (Savarese et al., 2022). HF is a complex cardiac condition characterized by a diminished heart capacity to pump sufficient blood or relax under normal left ventricle filling conditions, leading to symptoms of breathlessness, fatigue, and edema (McDonagh et al., 2021). It is classified into three clinical phenotypes according to left ventricular ejection fraction (LVEF) as reduced LVEF HF (HFrEF, LVEF≤40%), HFmrEF (LVEF between 40% and 49%), and preserved LVEF HF (HFpEF, LVEF≥50%) (McDonagh et al., 2021). Each phenotype requires specific treatment strategies, highlighting the importance of early and accurate diagnosis of HF phenotypes (McDonagh et al., 2021; McDonagh et al., 2024). For example, therapeutic strategies for HFrEF typically incorporate major classes of foundational therapies targeting specific mechanisms of action, such as renin-angiotensin system inhibitors [RASi, such as angiotensin-converting enzyme inhibitors (ACEIs), angiotensin II receptor blockers (ARBs), and angiotensin receptor-neprilysin inhibitors (ARNIs)], beta-blockers, mineralocorticoid receptor antagonists, and sodium-glucose transporter 2 (SGLT2) inhibitors as foundational therapies. In contrast, the approach for HFpEF is more conservative, focusing primarily on the management of symptoms with diuretics and comorbid conditions and the selective use of SGLT2 inhibitors, given their emerging evidence of benefit in this subgroup (McDonagh et al., 2024; Heidenreich et al., 2022). More importantly, early initiation and accelerated up-titration of HF therapy in the acute phase are associated with improved patient outcomes, and these benefits are likely to extend over longer-term clinical follow-up (Mebazaa et al., 2022; DeVore et al., 2020).

Cardiac ultrasonography is an essential tool for bedside LVEF assessment, though precise phenotypic classification from comprehensive echocardiography study is often performed late in the admission process (Bennett et al., 2022). This may delay the timely initiation of appropriate treatments specific to each HF phenotype and further limit the optimization and selection of pharmacological approaches under certain scenarios, for example, the intensive use of ARNI in AHF patients manifesting borderline hypotension. Recently, advancements in machine learning (ML) have led to significant progress in HF prediction models (Alotaibi, 2019; Toumpourleka et al., 2021; Tripoliti et al., 2016; Gallagher et al., 2019). Among these, random forests have been effective in the prediction of HF using a limited set of features (Ambale-Venkatesh et al., 2017), and the integration of diverse data types through recurrent neural networks and logistic regression has enhanced predictive accuracy (Chen et al., 2019). Nevertheless, most studies have focused on binary classification, primarily differentiating between HFrEF and HFpEF, with limited attention paid to comprehensive multiclass HF prediction, including HFmrEF (Ho et al., 2016; Mathis et al., 2020; Cherukupalli et al., 2022; Zhao et al., 2022). Additionally, the application of natural language processing (NLP) to HF prediction has been explored, demonstrating its effectiveness in extracting meaningful information from unstructured medical text records (Evans et al., 2016). These techniques have substantially improved the sensitivity of HF diagnosis and identification. Recent studies have also applied transformer-based NLP methods to EHR phenotyping with promising results (Shickel et al., 2022; Zhou et al., 2023; Zandbiglari et al., 2025). However, these approaches typically require very large training corpora and computational resources, which may limit their immediate applicability in many clinical settings.

Despite these advancements, a significant gap remains in providing a comprehensive HF prediction model encompassing all HF phenotypes, particularly when using data available early on hospital admission. Further, despite the high HF prevalence, the accurate diagnosis of AHF, particularly its clinical phenotypes, remains challenging. This study aimed to address this gap by developing an ML model that integrates both clinical narratives and laboratory test results obtained at the onset of hospital admission. While deep learning models such as CNNs and transformers have recently been applied to EHR phenotyping with promising results, these methods often require large-scale training data, substantial computational resources, and their interpretability in clinical practice remains limited. In contrast, we adopted a random forest-based approach to balance predictive performance with practicality. Random forest is computationally efficient, parallelizable, and cost-effective, while offering interpretable outputs through feature importance analysis. Although it may not always outperform deep learning models, our results demonstrate that it provides clinically acceptable accuracy for the multimodal prediction of acute HF phenotypes. By combining the NLP and ML techniques, we sought to facilitate the early prediction of HF phenotypes.

## 2 Methods

### 2.1 Data source

The Medical Information Mart for Intensive Care III (MIMIC-III) is a publicly available medical database that includes de-identified health-related data associated with over 40,000 patients who stayed in the intensive care units (ICUs) of the Beth Israel Deaconess Medical Center between 2001 and 2012 (Johnson et al., 2016). Despite its focus on ICU patients, the MIMIC-III database is suitable for HF research because of its extensive and diverse collection of critical care data encompassing a wide range of patient demographics and clinical details, which are essential for an in-depth analysis of HF phenotypes. It contains information, such as demographics, vital sign measurements over time, laboratory test results, procedures, medications, caregiver notes, imaging reports, and survival data (including dates and times).

### 2.2 Participants

The primary objective of this study was to predict HF phenotypes using the information available during the early stages of hospital admission. Patient data were retrospectively retrieved from the MIMIC-III database (original patient number = 58,976), focusing on those diagnosed with HF (original number = 2,509 patients), as indicated by relevant ICD-9 codes (Yancy et al., 2013). To ensure a focus on early prediction, only data from the first admission at which HF was diagnosed were included. Subsequent admissions of the same patient were excluded, with 1,954 non-repeated HF patient numbers, aligning with our objective of early phenotype detection.

### 2.3 Data types

This study utilizes two primary data types from the MIMIC-III database: structured and unstructured. The structured data encompassed a variety of biochemical test items, represented in tabular formats, with rows corresponding to individual patients and columns corresponding to various biochemical values, including blood glucose levels, cholesterol, and other laboratory test results. The unstructured data consisted of clinical narratives containing textual descriptions of patient conditions, diagnostic findings, and treatment plans, which were recorded in free text by medical professionals. Detailed data preparation, labeling, feature selection, and importance are provided in the Supplementary material.

### 2.4 LVEF labeling and HF phenotypes classification

**Figure 1:**
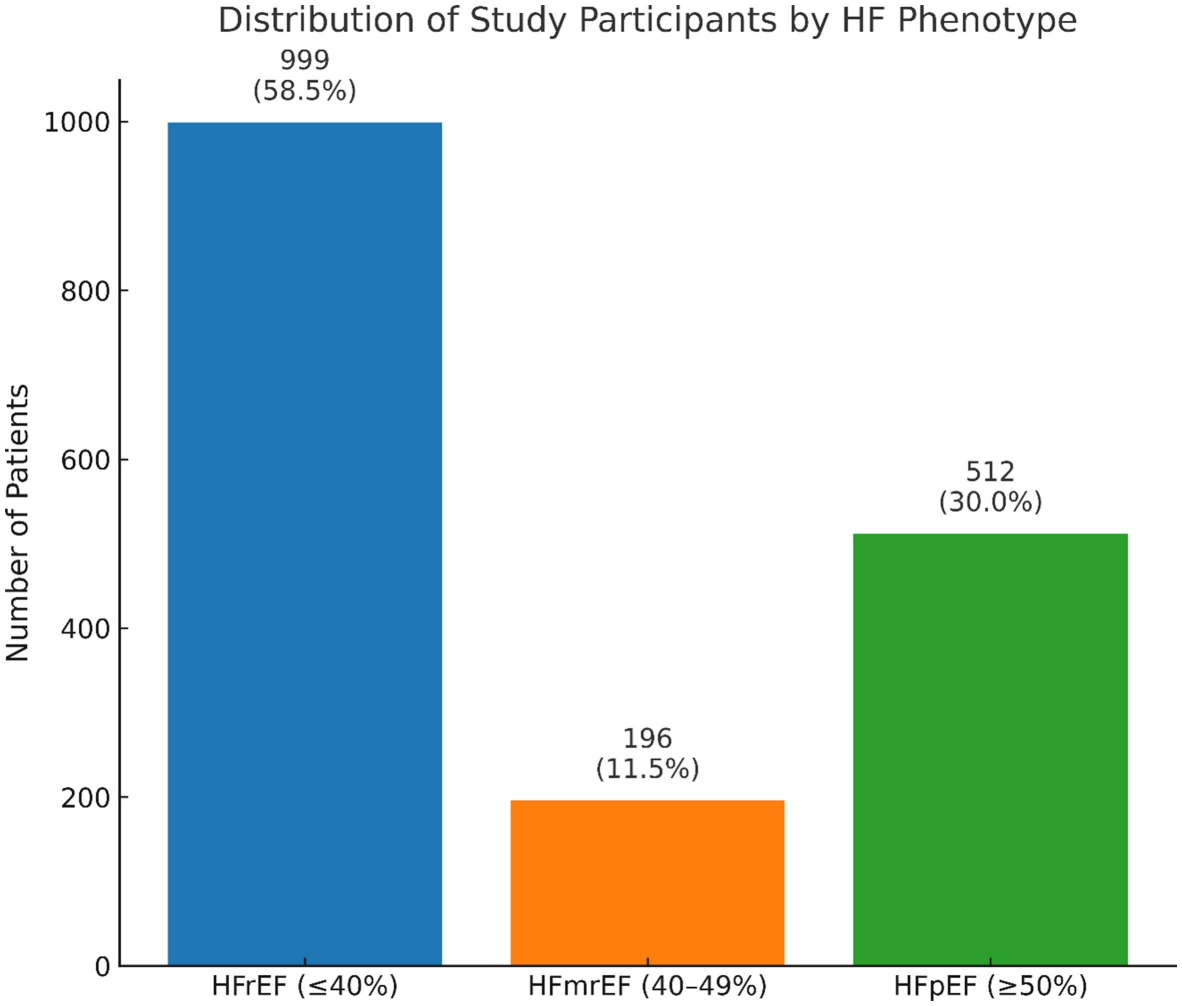
The distribution of HF phenotype classification displayed from final study participants with LVEF information available.

Detailed LVEF data cleansing information is provided in the Supplementary material. In the current study, HF phenotypes as major outcome measures were differentiated based on LVEF values, typically documented in the MIMIC-III database within cardiac ultrasound reports or data mentioned in nursing reports and discharge summaries. We extracted the text records of 1,954 patients with HF to identify LVEF values. In cases where multiple LVEF values were recorded for each patient during their hospital stay, only the first recorded LVEF value was used. This process resulted in the identification of 1,707 patients (247 omitted due to missing LVEF values) with accurately determined LVEF values for the categorized HF phenotypes (Supplementary Figure 1). Our final HF study participants were then labeled according to their respective HF phenotypes: 999 as HFrEF (LVEF≤40%), 196 as HFmrEF (LVEF>40%, <50%), and 512 as HFpEF (LVEF≥50%) (Figure 1).

### 2.5 Study design

The research workflow is illustrated in Supplementary Figure 1. Feature selection and data structuring play critical roles in the development of HF phenotype prediction models. Unstructured textual data and biochemical test data from the training dataset (*n* = 1,192) were subject to feature selection using the LASSO method. The feature selection process, detailed in the “Feature Selection and Data Processing” subsection in Supplementary material, identified 47 key textual features and 53 biochemical test features (Supplementary Tables 1, 2, Supplementary material) as the most relevant for our analysis. The selected textual features were converted into structured data using one-hot encoding. To eliminate the potential biased model training from cardiac ultrasound information, all relevant data, including those reported in the cardiac formal ultrasound report section or terms and any findings derived from ultrasound imaging reports (such as nursing reports and discharge summaries), were omitted from our model training. The study flowchart is reported in Supplementary Figure 1.

Using the prepared data, we constructed three variants of random forests: the first using only textual data, another using only biochemical data, and a third combining both data types. This approach allowed us to compare the performance of each model type in classifying HF phenotypes.

### 2.6 Statistics

For continuous variables, since at least one of the subgroups was not normally distributed, the Kruskal-Wallis test was used to assess differences among the three HF phenotypes, and the Chi-square test was used to determine statistical significance for categorical variables.

To ensure robustness and address the imbalances in the dataset, we employed a five-fold cross-validation method to train and validate the models. Additionally, SMOTE was used to balance the training data, thereby enhancing the validity of the cross-validation process. We then calculated the averages and standard deviations of various performance metrics across the five-fold cross-validation to assess the capabilities of each model comprehensively. The areas under the ROC and PRC curves (AUROC and AUPRC, respectively) were used to outline the overall diagnostic yield and overall performance of a classifier by focusing on the minority class (assuming *imbalanced* datasets). Finally, an independent validation was performed using a separate test dataset (*n* = 513). This step was crucial to confirm the generalizability of our models and check for potential over-fitting issues, ensuring that our models were reliable and applicable in real-world clinical settings.

All analyses were conducted using Python 3.9.13. Machine learning models, including random forest classifiers and LASSO feature selection, were implemented with scikit-learn version 1.2.2. Data balancing was performed using the SMOTE implementation from the imbalanced-learn package (version 0.10.1). Model interpretability analyses were performed using the shap package (version 0.41.0).

## 3 Results

The demographic characteristics of the patients by 3 HF phenotypes are shown in Table 1. The median age for patients with HFrEF was significantly younger, with male sex predominance (63.2%) compared with those with HFpEF and HFmrEF phenotypes (*p* = 0.04 and <0.01, respectively). Patients with the HFpEF phenotype had the highest systolic blood pressure and heart rate, followed by those with the HFmrEF and HFrEF phenotypes (*p* < 0.01). Racial distribution was not significantly different among the three groups. Ischemic cardiomyopathy was more prevalent in patients with HFrEF (21.66%) than in those with HFpEF (6.05%) or HFmrEF (12.76%) (*p* < 0.01). History of myocardial infarction and coronary artery disease was also more common in patients with HFrEF and HFmrEF than in patients with HFpEF (both *p* < 0.01). Conversely, valvular heart disease was more frequent in patients with HFpEF (66.02%) than in those with HFmrEF (60.71%) or HFrEF (54.76%) (*p* < 0.01).

**Table 1:** Comparison of characteristics among HF patients with preserved, mildly-reduced, and reduced ejection fraction.

Characteristic	HFpEF (*n* = 512)	HFmrEF (*n* = 196)	HFrEF (*n* = 997)	*p*-value
Age (years)	71.00 (58.00–80.00)	71.00 (57.00–80.00)	68.00 (56.00–78.00)	0.04
Male gender (%)	48.83%	63.27%	63.19%	<0.01
Systolic blood pressure (mmHg)	128.00 (110.00–147.00)	126.00 (110.00–140.00)	116.00 (103.00–134.00)	<0.01
Diastolic blood pressure (mmHg)	68.00 (58.00–80.00)	70.00 (57.00–80.25)	67.00 (58.00–77.00)	0.26
Heart rate (beats/min)	80.00 (66.00–92.00)	80.00 (65.00–96.00)	84.00 (70.00–100.00)	<0.01
Race/ethnicity				0.39
Non-Hispanic White (%)	70.70%	65.31%	67.10%	0.25
Non-Hispanic Black (%)	13.67%	11.73%	14.04%	0.69
Hispanic (%)	3.52%	5.10%	3.51%	0.54
Asian (%)	0.98%	3.06%	2.01%	0.14
Other (%)	11.13%	14.80%	13.34%	0.33
Etiology				
Ischemic cardiomyopathy (%)	6.05%	12.76%	21.66%	<0.01
Hypertensive heart disease (%)	7.03%	7.14%	7.42%	0.96
Dilated cardiomyopathy (%)	6.05%	9.69%	26.38%	<0.01
Valvular heart disease (%)	66.02%	60.71%	54.76%	<0.01
Arrhythmias (%)	87.89%	89.29%	86.66%	0.54
Comorbidities				
Hypertension (%)	56.64%	52.55%	55.97%	0.61
Hyperlipidemia (%)	34.96%	41.33%	35.51%	0.25
Diabetes (%)	23.05%	23.47%	27.28%	0.16
Prior myocardial infarction (%)	21.29%	31.63%	33.10%	<0.01
Peripheral vascular disease (%)	9.18%	10.71%	11.33%	0.44
Coronary artery disease (%)	39.06%	51.53%	49.65%	<0.01
Atrial fibrillation/flutter (%)	65.63%	71.94%	66.70	0.27
COPD or Asthma (%)	20.12%	18.88%	16.65%	0.24
Stroke/transient ischemic attack (%)	8.59%	7.65%	9.13%	0.79
Renal insufficiency (%)	22.85%	26.02%	20.66%	0.21
Chronic dialysis (%)	3.91%	5.61%	3.61%	0.42
CRT (%)	1.17%	1.02%	3.51%	<0.01
ICD (%)	6.25%	8.67%	19.96%	<0.01
Anemia (%)	19.73%	19.39%	16.15%	0.18
Symptoms and signs				
Dyspnea on exertion (%)	66.60%	63.78%	68.61%	0.37
Orthopnea (%)	8.20%	7.14%	11.94%	0.02
PND (%)	2.73%	3.06%	5.72%	0.02
Ankle edema (%)	43.36%	38.78%	43.33%	0.48
Rales (%)	22.07%	25.51%	25.78%	0.27
Third heart sound gallop (%)	1.37%	2.04%	2.41%	0.40
Neck vein distension (%)	9.77%	9.18%	12.64%	0.15
CXR findings				
Pulmonary edema (%)	29.04% (97/334)	26.06% (37/142)	28.53% (196/687)	0.80
Cardiomegaly (%)	45.81% (153/334)	54.23% (77/142)	60.55% (416/687)	<0.01
Pleural effusion (%)	40.12% (134/334)	45.77% (65/142)	42.50% (292/687)	0.51
Laboratory investigations				
Hemoglobin (mg/dl)	11,200 (9680–12,800)	11,550 (10480–12,900)	11,800 (10500–13,500)	<0.01
Serum sodium (mEq/L)	138.50 (136.00–141.00)	139.00 (136.27–141.00)	138.00 (135.50–140.00)	0.01
Serum potassium (mEq/L)	4.20 (3.80–4.61)	4.30 (3.90–4.70)	4.20 (3.90–4.65)	0.22
Creatinine (mg/dl)	1.19 (0.90–1.70)	1.20 (0.90–1.80)	1.20 (0.90–1.75)	0.21
Pharmacological treatment				
ACE inhibitors/ARBs (%)	17.19%	23.47%	22.57%	0.04
Beta blockers (%)	54.69%	60.20%	61.38%	0.04
MRA (%)	2.73%	3.06%	5.22%	0.05
Loop diuretics (%)	52.73%	56.12%	62.09%	<0.01
Statins (%)	20.51%	22.96%	22.37%	0.66
Antiplatelet agents (%)	19.14%	24.49%	23.17%	0.14
Anticoagulants (%)	51.56%	62.76%	58.48%	<0.01

COPD, chronic pulmonary obstructive disease; CRT, cardiac resynchronization therapy; ICD, implantable cardioverter-defibrillator; NYHA, New York heart association; PND, paroxysmal nocturnal dyspnea; ECG, electrocardiogram; LBBB, left bundle branch block; RBBB, right bundle branch block; CXR, chest x-ray; ACE, angiotensin-converting enzyme; ARB, angiotensin II receptor blocker; ARNI, angiotensin receptor-neprilysin inhibitor; MRA, mineralocorticoid receptor antagonist, SGLT2i: sodium glucose co-transporter 2 inhibitor.

Laboratory investigations revealed higher hemoglobin levels in patients with HFrEF (median 11,800 mg/dL) than in patients with HFpEF and HFmrEF (*p* < 0.01). Drug treatment patterns indicated that ACEIs or ARBs and beta-blockers were significantly more commonly used in patients with HFrEF and HFmrEF than in those with HFpEF (*p* = 0.04), whereas loop diuretics were most frequently prescribed to patients with HFrEF (62.09%) compared to HFpEF and HFmrEF (*p* < 0.01).

### 3.1 Performance of HF phenotype prediction models using different data configurations

Table 2 outlines the performance across different data configurations, including the accuracy, precision, recall, F1-score, and AUROCs, for models trained on textual data, laboratory data, and a combination of both. The training performance, as detailed in Table 2A, indicates that models using combined data achieved the highest performance, with an accuracy of 0.70 ± 0.03 and an AUROC of 0.76 ± 0.02. Models relying on textual data alone also performed well, demonstrating an accuracy of 0.69 ± 0.04 and an AUROC of 0.77 ± 0.03. In contrast, models based solely on laboratory data had lower accuracy and AUROC values of 0.50 ± 0.02 and 0.55 ± 0.02, respectively.

**Table 2:** Performance of HF phenotype prediction models using different data configurations.

A. Training Performance					
Model input	Accuracy	Precision	Recall	F1-score	AUROC
Textual data	0.69 ± 0.04	0.51 ± 0.04	0.50 ± 0.03	0.49 ± 0.03	0.77 ± 0.03
Laboratory data	0.50 ± 0.02	0.39 ± 0.02	0.38 ± 0.01	0.38 ± 0.02	0.55 ± 0.02
Combined data	0.70 ± 0.03	0.55 ± 0.05	0.50 ± 0.02	0.49 ± 0.02	0.76 ± 0.02

B. Independent test performance					
Model input	Accuracy	Precision	Recall	F1-score	AUROC
Textual data	0.71	0.67	0.51	0.51	0.78
Laboratory data	0.55	0.31	0.34	0.31	0.55
Combined data	0.73	0.65	0.51	0.50	0.80

Independent test performance replicated these findings (Table 2B). The combined data models sustained their lead with an accuracy of 0.73 and an AUROC of 0.80. Textual data models followed closely with an accuracy of 0.71 and an AUROC of 0.78. Laboratory data models remained the least effective in this independent evaluation, with an accuracy of 0.55 and an AUROC of 0.55.

**Figure 2:**
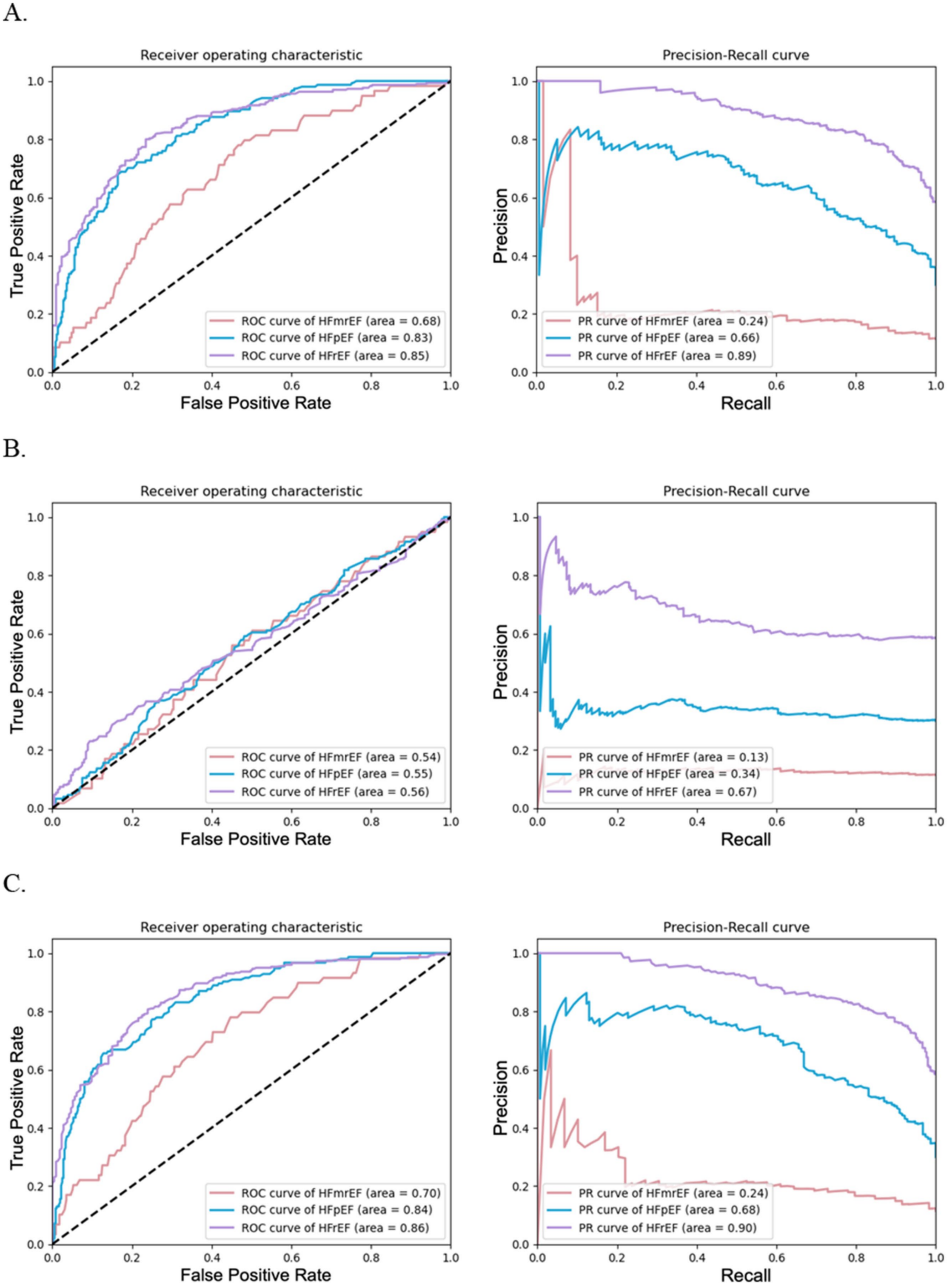
Performance of random forests for HF phenotype classification using different data configurations. **(A)** illustrates the effectiveness of random forest models using textual data with 47 features, **(B)** depicts models using laboratory test data with 53 features, and **(C)** shows models combining textual and laboratory test data with 100 features. Each subfigure includes ROC curves on the left and PRC curves on the right.

Figure 2 complements these results by presenting ROC and PRC curves for the binary classification of each HF phenotype. Figure 2A shows that models using textual data with 47 features had an AUROC of 0.68 for HFmrEF, 0.83 for HFpEF, and 0.85 for HFrEF, with corresponding AUPRCs as 0.24 for HFmrEF, 0.66 for HFpEF, and 0.89 for HFrEF, indicating a respectable performance, particularly for HFpEF and HFrEF phenotypes. Figure 2B represents models using laboratory data with 53 features and revealed lower performance, with AUROCs ranging from 0.54 to 0.56 and AUPRCs from 0.13 to 0.67. Figure 2C illustrates that models utilizing combined data with 100 features performed best, with AUROCs of 0.70 for HFmrEF, 0.84 for HFpEF, and 0.86 for HFrEF; corresponding AUPRCs were 0.24 for HFmrEF, 0.68 for HFpEF, and 0.90 for HFrEF, suggesting that the integration of data types enhanced the model’s predictive capabilities.

### 3.2 Performance of HF phenotype prediction models with reduced features

To assess the impact of feature reduction on the model performance, the models were streamlined to use subsets of the original features: 23 textual features, 26 laboratory test features, and a combination of both, totaling 50 features (32 textual and 18 test items).

Table 3 presents the performances of these reconfigured models in both the training and independent testing scenarios. Table 3A shows models utilizing the combined reduced features demonstrated superior performance from the training dataset, achieving an accuracy of 0.70 ± 0.04 and an AUROC of 0.76 ± 0.02. Models trained using only textual data also exhibited commendable performance, whereas those based solely on laboratory data had comparatively lower metrics.

**Table 3:** Performance of HF phenotype prediction models with reduced features using different data configurations.

A. Training performance					
Model input	Accuracy	Precision	Recall	F1-score	AUROC
Textual data	0.68 ± 0.02	0.52 ± 0.03	0.50 ± 0.01	0.50 ± 0.02	0.75 ± 0.01
Laboratory data	0.50 ± 0.02	0.39 ± 0.02	0.38 ± 0.01	0.38 ± 0.02	0.54 ± 0.02
Combined data	0.70 ± 0.04	0.52 ± 0.08	0.51 ± 0.04	0.50 ± 0.05	0.76 ± 0.02

B. Independent test performance					
Model input	Accuracy	Precision	Recall	F1-score	AUROC
Textual data	0.69	0.53	0.49	0.48	0.75
Laboratory data	0.56	0.32	0.35	0.32	0.55
Combined data	0.73	0.74	0.52	0.52	0.80

The independent test dataset results shown in Table 3B further substantiated these outcomes. Combined data models retained their lead in performance, with an accuracy of 0.73 and an AUROC of 0.80. The performance of models using textual data was closely followed, and laboratory data models, although least effective, showed results consistent with their training performance.

The binary classification performance of these models for the HF phenotypes was shown in Supplementary Figure 2. Supplementary Figure 2A shows the substantial capacity for phenotype differentiation using textual data, particularly for HFpEF (AUROC of 0.80 and of AUPRC 0.61) and HFrEF (AUROC of 0.83 and of AUPRC 0.87). Supplementary Figure 2B shows that models with laboratory data exhibit lower performance across all HF phenotypes, with AUROCs ranging from 0.53 to 0.57 and AUPRCs from 0.12 to 0.67. Supplementary Figure 2C confirms a balanced combination of 50 features demonstrating the best overall performance with the highest AUROC (ranging from 0.73 to 0.85) and AUPRC (ranging from 0.27 to 0.89) values for all HF phenotype.

### 3.3 Evaluating model performance with varying feature quantities

**Figure 3:**
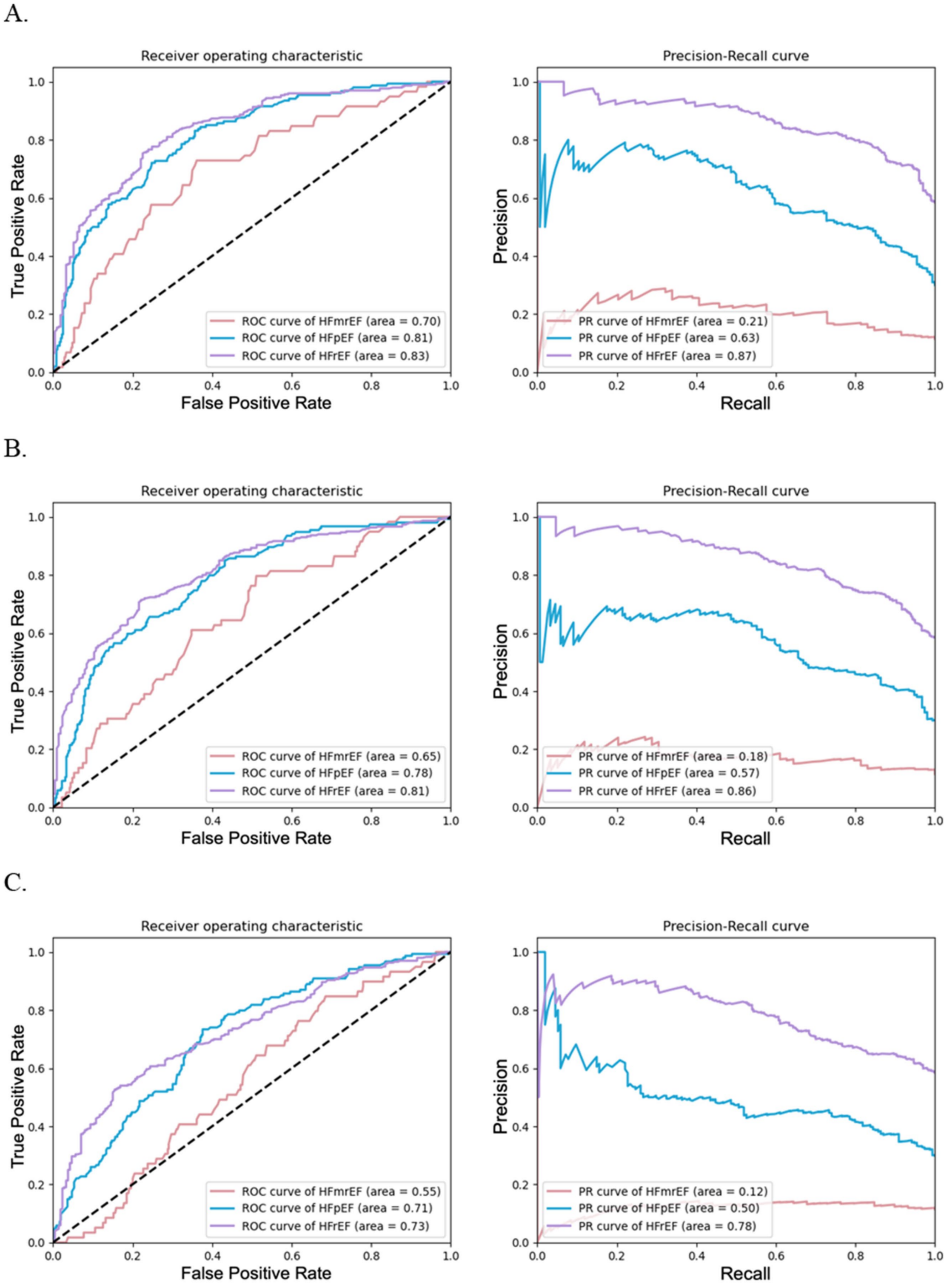
Performance of random forests for HF phenotype classification with varying feature quantities using combined data. **(A)** illustrates the effectiveness of random forest models using top 30 features, **(B)** depicts models using top 20 features, and **(C)** shows models using top 10 features. Each subfigure includes ROC curves on the left and PRC curves on the right.

We observed that random forests built with the top 50 to top 20 features exhibits comparable performance in the three-class classification of HF phenotypes, as shown in Table 4. The models maintained robustness, as evidenced by the minimal variation in the performance metrics during cross-validation and independent testing. The model with the top 20 features showed an accuracy of 0.67 ± 0.01 and an AUROC of 0.74 ± 0.01. However, the top 10 features model dropped to an accuracy of 0.54 ± 0.01 and an AUROC of 0.64 ± 0.02. The overall performances of random forests for each individual HF phenotype classification with varying feature quantities using combined data were displayed in Figure 3.

**Table 4:** Performance of HF phenotype prediction models with varying feature quantities using combined data.

A. Training performance					
Feature quantity	Accuracy	Precision	Recall	F1-score	AUROC
50	0.70 ± 0.04	0.52 ± 0.08	0.51 ± 0.04	0.50 ± 0.05	0.76 ± 0.02
30	0.66 ± 0.03	0.49 ± 0.05	0.49 ± 0.03	0.48 ± 0.04	0.74 ± 0.04
20	0.67 ± 0.01	0.55 ± 0.01	0.53 ± 0.01	0.53 ± 0.01	0.74 ± 0.01
10	0.54 ± 0.01	0.43 ± 0.01	0.44 ± 0.01	0.43 ± 0.01	0.64 ± 0.02

B. Independent test performance					
Feature quantity	Accuracy	Precision	Recall	F1-score	AUROC
50	0.73	0.74	0.52	0.52	0.80
30	0.69	0.45	0.48	0.46	0.75
20	0.69	0.45	0.48	0.46	0.75
10	0.63	0.40	0.45	0.42	0.68

The binary classification results for individual HF phenotypes depicted in Figure 3 and previously in Supplementary Figure 2C reinforce these findings. Figures 3A–C shows a decline in the area under the ROC and PRC curves as the number of features decreased for the models with the top 30, 20, and 10 features, respectively. This highlights the importance of retaining a critical mass of features to maintain predictive accuracy down to the number of top 20 features yet diminishing significantly with only the top 10 features. These data suggest a trade-off for accurate HF phenotype classification using the top 20 features that balance model accuracy and computational efficiency.

### 3.4 Feature importance analysis in predictive model performance and additive value distributions

**Figure 4:**
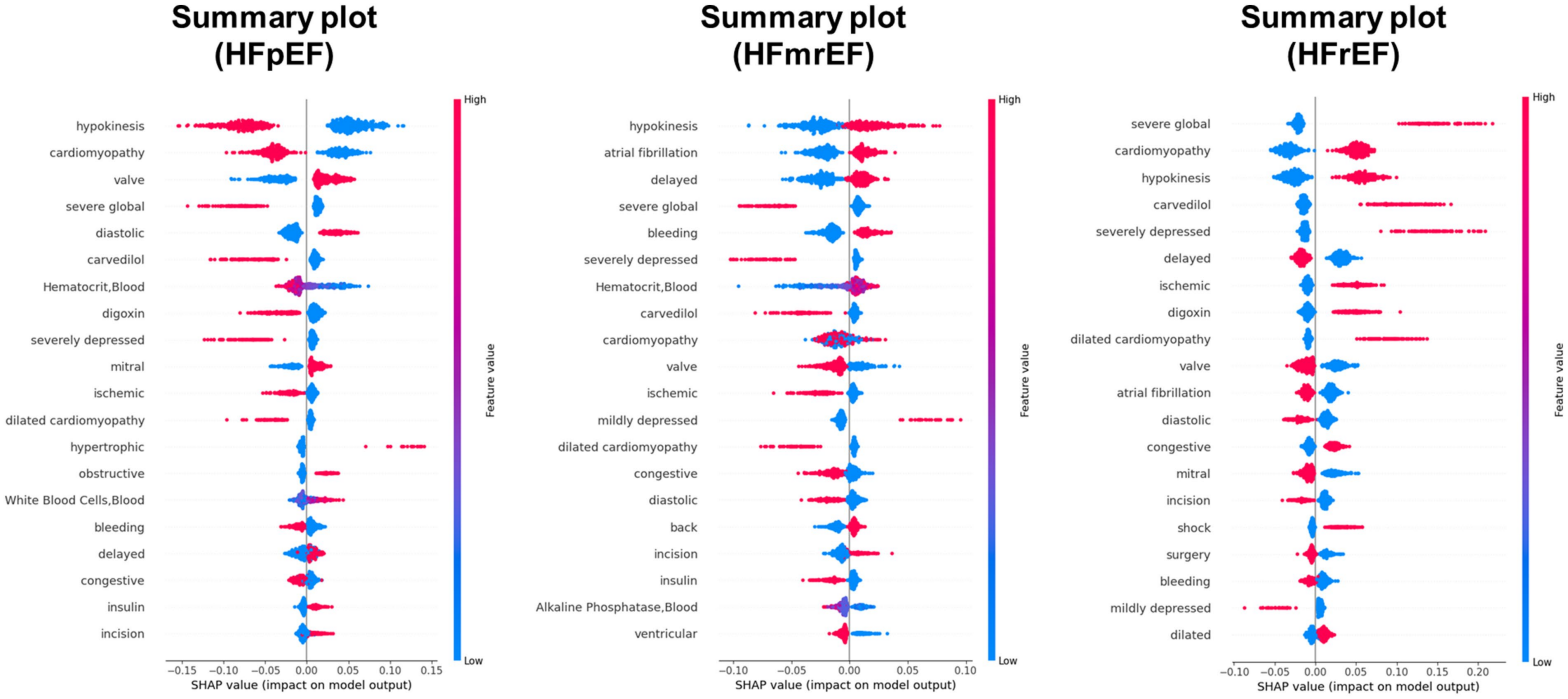
SHAP (SHapley Additive exPlanations) value distributions for top features in predicting HF phenotype. The figure presents summary plots for HFpEF (left), HFmrEF (middle), and HFrEF (right) phenotypes. Each row represents a feature, and each point represents a patient. The x-axis shows the SHAP value, indicating the impact of the feature on the model output. Red points denote high feature values, while blue points indicate low values. Features are ranked by their overall importance in predicting each phenotype. This visualization reveals the complex relationships between features and their impact on heart failure phenotype classification, highlighting key predictors such as hypokinesis, cardiomyopathy, and severe global dysfunction across different phenotype.

**Figure 5:**
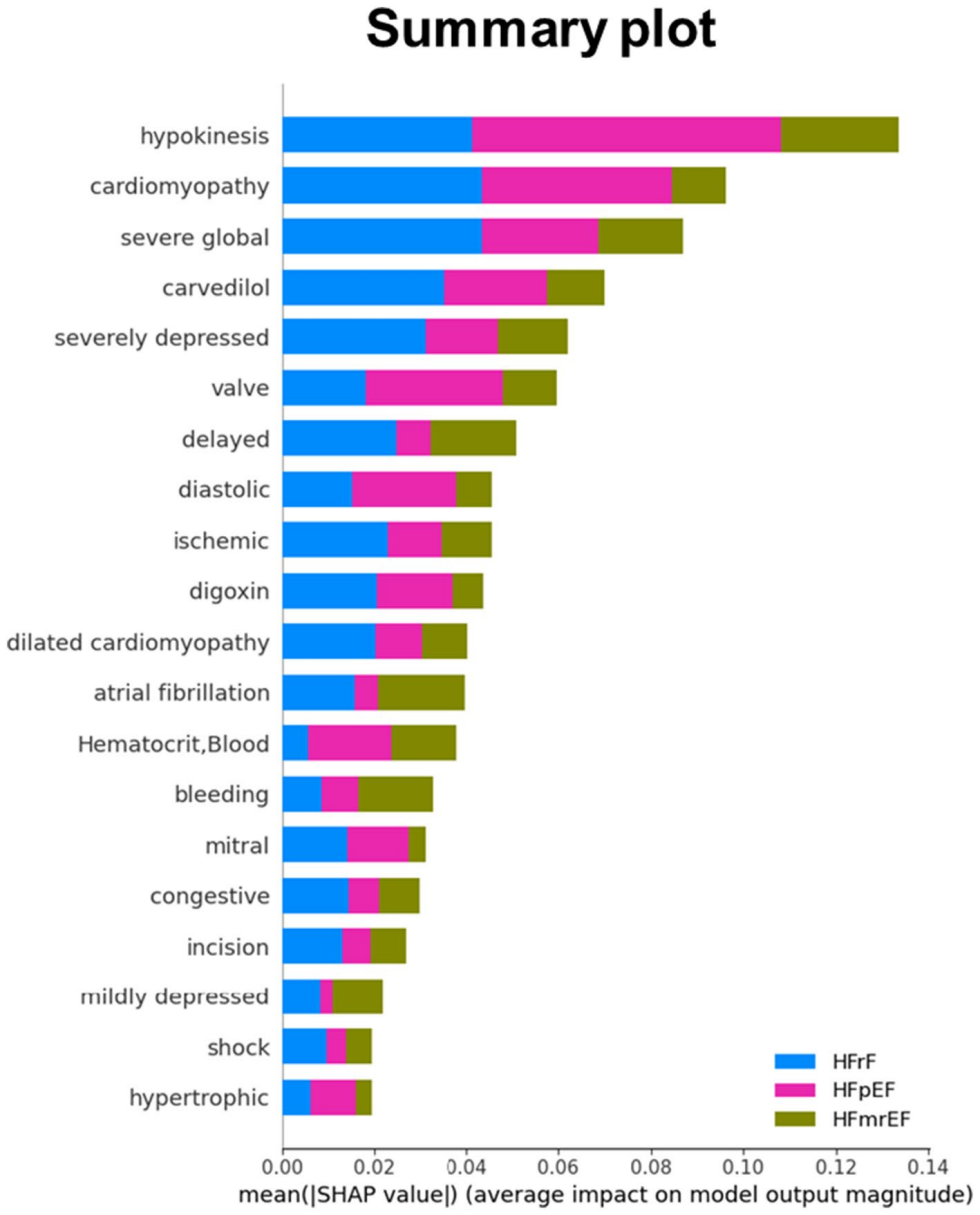
Mean SHAP (SHapley Additive exPlanations) values for top features across HF phenotype. This bar plot illustrates the average impact of key features on model output for HFrEF (blue), HFpEF (pink), and HFmrEF (olive) classifications. Features are ranked by their overall importance, with hypokinesis, cardiomyopathy, and severe global dysfunction showing the highest mean SHAP values. The x-axis represents the mean absolute SHAP value, indicating the magnitude of each feature’s impact on model predictions. This visualization provides a comparative view of feature importance across the three heart failure phenotypes, highlighting the differential influence of clinical and physiological factors in predicting each phenotype.

A detailed examination of the top 10 features across the five different models was conducted to analyze the impact of feature quantity on the performance of our HF phenotype predictive models. These models varied in the number of features used, ranging from the complete 100 features to a reduced set of 10. The feature importance based on 23 textual features, 26 laboratory test features and a balanced combination of 50 features (with 32 textual and 18 test items) was displayed in Supplementary Figures 3A–C. The SHapley Additive exPlanations (SHAP) value distributions and summary plot for top features based on 100 features model were further presented in Figures 4, 5, respectively.

## 4 Discussion

Our investigation into early AHF phenotype prediction led to the key finding that the integration of clinical text data and laboratory results significantly enhanced the accuracy of the AHF phenotype classification of HFrEF, HFmrEF, and HFpEF. Our approach, which employs both NLP and ML from clinical information and traditional laboratory data, underscores the value of combining diverse data sources into successful clinical diagnostics. By leveraging NLP alongside traditional laboratory analyses, our models tap into the rich narrative of clinical notes, capturing key messages that laboratory results alone might miss when distinguishing between HF phenotypes. The superior performance of our combined data models, particularly in terms of accuracy and AUROC, highlights the potential of multimodal data integration for improving diagnostic tools for AHF phenotypes, even with reduced features. This approach aligns with the growing trend in personalized medicine using ML models, where detailed information is vital for precisely predicting the AHF phenotype.

Our exploration of feature reduction and model performance revealed critical insights with practical implications. The models retained high accuracy and AUROCs, even when the feature count was markedly reduced. This suggests that a well-selected subset of features can be as effective as a complete set, thus highlighting the efficiency of our models. Notably, there is a critical point when reducing feature numbers to 10, where further reduction significantly impacts the model efficiency, indicating a threshold below which the model can no longer effectively capture the complexity of HF phenotype distinctions. Balancing accuracy and computational efficiency is vital for developing practical HF prediction tools in diverse clinical environments. Our findings have several significant clinical implications. The accurate and timely classification of HF phenotypes has the potential to revolutionize early patient management and to guide timely mechanism-driven treatment strategies balancing treatment benefits, expenditure, and overall adverse effects caused. For example, early initiation and more intensive use of RASi, MRA, or beta-blockers critical HFrEF patients for evidence based top priority (Class I) (McDonagh et al., 2021; Heidenreich et al., 2022; DeVore et al., 2020; Velazquez et al., 2018; Gottlieb et al., 2002; Zannad et al., 2011) with rapid onset of efficacy on survival, especially when more considerations needed, compared to sGLT2 inhibitor alone for the HFpEF population. This precision in diagnosis could facilitate more targeted therapies, align treatment plans with individual patient profiles, and potentially alter the disease course.

In our analysis, “hypokinesis,” “dilated cardiomyopathy,” “severe global,” “severely depressed,” and “cardiomyopathy” emerged as pivotal textual features for HFrEF phenotype prediction, underpinned by pharmacological uses including “digoxin” and “carvedilol.” Terms of “hypokinesis” and “severely depressed” indicative of diminished myocardial contractility often suggests advanced HF stage, especially in HFrEF (Schmidt-Ott and Ascheim, 2006; Berezin et al., 2021). Similarly, “cardiomyopathy,” particularly the dilated form, involves certain inner morphological and functional myocardial anomaly (Seferović et al., 2019). For example, mutations in genes encoding sarcomere proteins, such as the beta-myosin heavy chain, have been associated with the development of dilated cardiomyopathy (Kamisago et al., 2000), underscoring the genetic underpinnings that contribute to the critical textual features observed in our study. On the contrary, terms of “diastolic” may provide a clue to the presence of diastolic anomaly delineating HFpEF pathophysiology. Laboratory test features also delineated the physiological disturbances in HF. For example, alterations in hematocrit or white blood cell count may reflect systemic anemic status relating to iron deficiency, chronic kidney disease or chronic inflammation process closely linked to HFpEF pathophysiology (Melenovsky et al., 2016; Loncar et al., 2021). The presence of leukocytosis (elevated white blood cells) indicated the pro-inflammatory status aligned with HFpEF central pathophysiology (Briasoulis et al., 2016; Yndestad et al., 2006). The constellation of these features supports the heterogeneity of HF and emphasizes the importance of a comprehensive approach to classify and manage this complex syndrome accurately.

In the realm of HF phenotype prediction, our study distinguished itself by integrating clinical text and laboratory data, in contrast to several other notable studies in the field. Alkhodari et al. made significant strides by employing deep learning to predict LVEF from patient clinical profiles and categorized HF into different LVEF cutoffs (Alkhodari et al., 2021). Their innovative approach utilizes LVEF ranges that differ from those of most contemporary classifications. This distinction highlights the challenge of a direct comparison, although both studies underscore the growing role of advanced computational methods in HF diagnosis. Uijl et al. focused on identifying HF LVEF phenotype using logistic regression models with routine clinical characteristics (Uijl et al., 2020). Their results were particularly strong in predicting HFpEF and HFrEF but less so for HFmrEF. Desai et al. developed a Medicare claims-based model to predict LVEF classes in patients with HF by leveraging administrative data (Desai et al., 2018). While valuable in health service research, these studies did not address the critical need for HF phenotype detection during the acute admission phase. Our study fills this gap by leveraging NLP and ML, offering a pathway for phenotype-specific early and precise administration of medications (Heidenreich et al., 2022; Velazquez et al., 2018; Voors et al., 2022). This is particularly crucial given the considerable time and expense associated with cardiac ultrasound, which is currently the standard for accurate HF phenotype prediction. Collectively, these prior studies demonstrate the growing interest in computational approaches for HF characterization, yet they differ substantially in objectives, data sources, and outcome definitions. To our knowledge, no existing model has sought to predict all three HF phenotypes simultaneously during the acute admission phase. Therefore, direct quantitative comparison is not possible, but our work provides an important benchmark in this emerging area by uniquely integrating both clinical text and laboratory data for early phenotype-specific prediction.

An important observation is that the performance of our model was lower for HFmrEF (AUROC = 0.70) compared with HFrEF (AUROC = 0.86) and HFpEF (AUROC = 0.84). This finding is consistent with clinical experience, as HFmrEF is often regarded as a heterogeneous and transitional phenotype with overlapping features of systolic and diastolic dysfunction. The ambiguity of its pathophysiological profile likely contributes to its reduced predictability. Future refinement of prediction models, potentially incorporating additional biomarkers or longitudinal trajectories, will be necessary to improve discrimination of this intermediate phenotype.

Although our models achieved robust AUROC values across all feature sets (Tables 2–4), precision and recall were relatively modest. This likely reflects the residual effects of class imbalance, which remains a well-recognized challenge in multiclass heart failure prediction. These findings suggest that while our approach is effective in discriminating phenotypes overall, additional strategies for imbalance correction may be needed to optimize case-level detection performance.

### 4.1 Limitations

Although our study provides valuable insights into the early prediction of the HF phenotype, it is important to acknowledge its limitations. The extensive use of the MIMIC-III database may limit the generalizability of our findings across different patient demographics and healthcare settings. Specifically, the MIMIC-III database predominantly consists of patients with AHF rather than ambulatory or non-ICU population. This distinction is crucial because the dynamics and characteristics of acute HF may differ significantly from those of chronic HF or community-managed cases, potentially affecting the applicability of our predictive models. Therefore, future research should include external validation on diverse cohorts, particularly non-ICU and outpatient populations, to confirm the robustness and clinical utility of our approach. Expanding beyond MIMIC-III to incorporate a broader range of patient populations, additional laboratory data (e.g., natriuretic peptides), and multimodal sources such as imaging and physiological signals will further enhance prediction accuracy and generalizability. Additionally, exploring the implementation and impact of these models in real-world clinical practice is essential for assessing their practical utility and effectiveness in patient care.

Another limitation relates to dataset labeling: 247 patients were excluded due to missing LVEF values, which were necessary for assigning ground-truth HF phenotypes during training. Importantly, this issue pertains only to retrospective dataset construction and not to the real-world use of our prediction model. In clinical deployment, our model does not require LVEF as input and can operate using only text and laboratory features. Thus, while missing LVEF reduced the training sample size in this study, it does not hinder the practical applicability of the model in real-world settings. Moreover, the generalizability of our findings may be limited by the ICU-based population in the MIMIC-III dataset. Validation in multicenter cohorts and in non-ICU and outpatient populations will be essential to confirm external applicability.

## 5 Conclusion

This study successfully developed a random forest model using clinical text and laboratory data from the MIMIC-III database for early prediction of all three HF phenotypes. Our approach, which combines textual features with laboratory test data, showed enhanced predictive accuracy, marking a significant contribution to HF diagnostics. By potentially initiating and guiding tailored treatment decisions during early admission, our AI model stands to improve the clinical outcomes of patients with HF markedly. The integration of diverse data types not only strengthens the model’s performance but also aligns with contemporary trends in personalized medicine. Furthermore, our exploration of feature reduction revealed the potential for efficient yet effective predictive models, emphasizing the balance between model complexity and clinical applicability. Future efforts should focus on refining the feature set, exploring additional data types, and integrating the model into actual clinical settings to broaden its utility in HF management.

### 5.1 Clinical perspectives

Our current work highlights the potential for early discrimination of diverse HF phenotype based on text and laboratory features blinded to imaging (such as cardiac ultrasound) information during acute phase with acceptable accuracy. This may facilitate timely delivery of treatment interventions that may balance efficacy and risk ratio for patients. As evidence increasingly demonstrates persistent clinical benefits of earlier and more intensive use of trial-proven medications among the AHF population, our data suggest that aggressive delivery of mechanism-specific foundational therapy can be initiated without time delay after balancing the consequences of adverse events, overall expense, and gain of survival (e.g., RASi or MRA among HFrEF with borderline hypotension). Our prediction model therefore provides an opportunity for precision medicine extended to a more tailored therapy decision among HF patients during the acute phase.

## Data Availability

The raw data supporting the conclusions of this article will be made available by the authors, without undue reservation.
