# Supplementary material for: Machine learning enhanced acute heart failure phenotype prediction using natural language processing and random forest
Source: Front Artif Intell. 2025 Oct 16;8:1664627. doi: 10.3389/frai.2025.1664627 (PMC12571787; doi:10.3389/frai.2025.1664627)
Supplement: Supplementary file 1 [file Data_Sheet_1.docx]

Supplemental Materials

**International Statistical Classification of Diseases (ICD) Coding for HF**

ICD-9 Codes (International Statistical Classification of Diseases and Related Health Problems) used for MIMIC-III database HF diagnosis in current study: 4254, 4255, 4256, 4257, 4258, 4259, 428, 39891, 40201, 40211, 40291, 40401, 40403, 40411, 40413, 40491, and 40493

**LVEF Data Extraction**

Given the varied representation of LVEF (e.g., LVEF, lvef, ejection fraction, ef, or EF), all text records were converted to lowercase, and regular expressions were used to extract the relevant terms and their corresponding values. However, LVEF values are not structured tabular data but are recorded in free text by medical staff, leading to potential inconsistencies in notation. Thus, each extracted value was manually verified for accuracy.

**Data Types**

This study utilized two primary data types from the MIMIC-III database: “structured” and “unstructured”. The “structured data” encompassed a variety of biochemical test items, represented in tabular formats, with rows corresponding to individual patients and columns to various biochemical values such as blood glucose levels, cholesterol, and other laboratory test results. The “unstructured data” consisted of clinical narratives containing textual descriptions of patient conditions, diagnostic findings, and treatment plans, recorded in free text by medical professionals.

**Data Cleansing and Preparation**

***Structured Data Cleansing***

For structured data, which consisted of biochemical tests conducted on patients, we extracted the test data for these 1,707 patients on the day of admission. Any abnormal values were identified and verified with collaborating cardiologists. For example, a patient with a creatinine value of 808 mg/dL, which far exceeds normal human limits, was excluded from the dataset. If a patient had undergone multiple tests of the same kind on the same day, the average of these values was used to represent the patient’s status for that test item. Not all patients underwent tests on their day of admission, resulting in the exclusion of patients without admission day test data. Consequently, the number of patients with usable structured data was reduced to 1,639 with 261 test items.

***Unstructured Data Cleansing***

The unstructured data comprised free-text medical records, primarily admission summaries. Since MIMIC-III does not record admission summaries, we used discharge summaries to simulate the content of admission summaries. These summaries included patient information such as medical history, social history, family history, and physical examination details recorded at the time of admission. Additionally, all previous discharge summaries of the patient before their first HF diagnosis were also extracted.

In cleaning the unstructured data, we first identified and removed any duplicates or incomplete records. Text data that were intended to simulate early admission summaries were cleansed of terms directly related to the diagnosis or LVEF values, such as “lvef,” “heart failure,” and “ejection fraction,” to mimic the scenario where the patient had not yet been diagnosed with HF. Titles and frequently occurring drug-related units were also removed to prevent interference in subsequent textual feature selection analysis. After these cleansing steps, we retained text records for 1688 patients.

***Feature Selection and Data Processing***

The dataset for this study was composed by merging structured and unstructured data corresponding to 1,639 and 1,688 patients, respectively, from the MIMIC-III database. This integration yielded a cohort of 1705 unique HF patients. To prepare the dataset for model development, stratified random sampling was employed to partition the data into training (70%) and test (30%) sets. This partitioning technique ensured proportional representation of the three HF subtypes, including HFrEF, HFmrEF, and HFpEF, across both sets. Such stratification is instrumental in preserving the dataset’s integrity and ensuring that the training and testing phases are conducted on balanced and representative samples.

The training set was instrumental for all downstream processes, including feature selection and model training, enabling the model to learn the patterns associated with each HF subtype. The test set was exclusively utilized for the final model evaluation, serving as a benchmark for assessing the model’s predictive performance and its ability to generalize to new, unseen data.

***Structured Data Feature Selection***

The study initially identified 261 biochemical test items in the training dataset. Some of these items were not tested for any patients and were thus excluded, leaving 245 items. Not every patient underwent all 245 tests, resulting in missing values across various items. These missing values were imputed using the median value of each item within the training dataset.

The LASSO (Least Absolute Shrinkage and Selection Operator) method was employed to refine the feature set further. [1] By setting the regularization parameter in LASSO to 0.1, certain feature coefficients were reduced to zero. Features with zero coefficients were deemed non-contributory to the model and thus were excluded from the final feature set. This process resulted in a condensed list of 53 critical biochemical test items (**Supplemental Table 1**), which were used as features for the structured data in the model.

***Unstructured Data Feature Selection***

For unstructured data comprising clinical narratives, we first excluded patients without textual records, yielding 1,178 text documents. These documents were then vectorized using the Term Frequency-Inverse Document Frequency (TF-IDF) method [19]. TF-IDF is a two-part process: the first part, Term Frequency (TF), represents the frequency of a word in a document. The second part, Inverse Document Frequency (IDF), inversely weights words based on their frequency across all documents, thus highlighting the uniqueness of words.

After calculating TF-IDF for each word in our documents, we obtained a vector representation for each document. In this study, we set a maximum feature number of 100,000 and used an n-gram range from 1 to 3, considering combinations of one, two, and three words. The vectorized text data were then subjected to LASSO with a regularization parameter of 0.0005. This process identified 47 textual features related to HF, such as “atrial fibrillation,” “cardiomyopathy,” and “coronary artery disease.”

Finally, to facilitate subsequent analysis, the unstructured text data were converted into structured data. Using one-hot encoding, the selected 47 words were transformed into 47 columns in a structured data table (**Supplemental Table 2**). Each patient’s text data was reviewed to determine the presence of these 47 words. If a word appeared in a patient’s text, the corresponding column was marked as 1; otherwise, it was marked as 0.

**Feature Importance**

In developing our predictive models, assessing the importance of input features was paramount. Utilizing the random forest algorithm, we analyzed how individual features influenced the model’s predictions. The Gini impurity index was leveraged to measure the effectiveness of features in separating different HF subtypes. [2] This criterion, intrinsic to decision trees within the random forest, calculates the impurity reduction each feature contributes when creating tree splits.

We quantified the average decrease in Gini impurity across all decision trees in the ensemble, standardizing this value to range between 0 and 1. Consequently, features that led to a more significant decrease in Gini impurity were considered more impactful as they provided more substantial differentiation between the classes.

For all the methods used in our study, including data preprocessing and model construction, we employed Python 3.9.13 and scikit-learn 1.2.2.

**Methodological Framework for Model Development**

***Data Normalization***

To address the challenge of varying units and value ranges in our dataset, we implemented z-score standardization for data normalization. This method transforms the data into a standard normal distribution with a mean of zero and a standard deviation of one, ensuring uniform influence of each feature in the model. Notably, the test set was normalized using the mean and standard deviation from the training set to prevent information leakage and maintain the integrity of our evaluation of unseen data.

***Handling Data Imbalance***

Given the varied prevalence of HF subtypes in the MIMIC-III database, balancing the dataset was crucial to avoid model bias. We employed the Synthetic Minority Over-sampling Technique (SMOTE) to generate synthetic samples for underrepresented classes, enhancing the model’s performance across all HF subtypes [20]. SMOTE was applied to each fold of the training set during cross-validation, fostering the development of a generalizable model.

***Machine Learning Model***

The random forest was selected for its robustness and ability to handle complex data types without over-fitting [3]. We constructed a model with 500 trees, optimizing for computational efficiency and pattern recognition. To accommodate the multiclass nature of HF subtype prediction, we employed a one-vs-rest approach, extending the binary classification capability of random forests to our multiclass scenario. The model assigns a subtype to each patient based on the highest confidence level across individual binary classifiers, ensuring precise classification.

***Model Training and Validation***

We adopted a five-fold cross-validation technique for model training and validation to ensure rigor and prevent data leakage. This method partitions the data into subsets for training and validation, cycling through each subset to validate the model’s performance. Combined with the SMOTE technique, cross-validation provided a balanced training environment and a comprehensive evaluation of the model’s performance in HF subtype prediction.

***Performance Measures in HF Subtype Prediction***

In evaluating the predictive models for HF phenotype (HFrEF, HFpEF and HFmrEF), our study utilized a suite of performance metrics to provide a multidimensional view of accuracy and reliability. Fundamental to these metrics are the classifications of true positives (TP), where the model correctly predicts a specific subtype; false positives (FP), where a condition is incorrectly predicted as a particular subtype; true negatives (TN), which reflect accurate predictions of a subtype’s absence; and false negatives (FN), denoting a missed true subtype.

Based on these categories, we employed the following macro measures for a comprehensive assessment:

- ***Accuracy***: The ratio of correctly predicted instances (TPs and TNs) to the total number of cases, averaged across all HF subtypes.
- ***Precision***: The proportion of correct positive predictions for a specific subtype, calculated as TPs divided by the sum of TPs and FPs, then averaged across subtypes.
- ***Recall (Sensitivity)***: The ability of the model to correctly identify all positive instances of each subtype, expressed as TPs divided by the sum of TPs and FNs, averaged across subtypes.
- ***F1-Score***: The harmonic mean of precision and recall, calculated for each subtype and then averaged, offering a balanced view of model performance.
- ***AUROC (Area under the Receiver Operating Characteristic Curve)***: This metric gauges the model’s ability to differentiate each subtype from the rest, with an average value computed across all subtypes.
- ***AUPRC (Area under the Precision-Recall Curve)***: A measure of the trade-off between precision and recall for each subtype, averaged to give an overall performance metric.

**Supplemental Tables**

**Supplemental Table 1**: Feature selection fields for examination Data. Inspection items for feature selection. 53 important features were selected from the examination data using the LASSO method.

| Absolute CD3 Count | Gamma Glutamyltransferase | RBC, CSF,Cerebrospinal Fluid (CSF) |
| --- | --- | --- |
| Absolute Lymphocyte Count | Glucose | RBC, Other Fluid |
| Alanine Aminotransferase (ALT) | Glucose | RBC, Pleural |
| Albumin/Creatinine, Urine | Granulocyte Count | RBC,Urine |
| Alkaline Phosphatase | Haptoglobin | Sodium, Urine |
| Alveolar-arterial Gradient | Hematocrit | Tidal Volume |
| Amylase | Hyaline Casts | Triglycerides |
| Anion Gap | Immunoglobulin G | Urea Nitrogen, Urine |
| Asparate Aminotransferase (AST) | Iron Binding Capacity | Vitamin B12 |
| Bands,Blood | Lactate Dehydrogenase (LD) | WBC, Ascites |
| C-Reactive Protein | Lipase | WBC, Pleural |
| Calculated Total CO2 | Lymphs,Cerebrospinal Fluid (CSF) | White Blood Cells |
| Chloride | NTproBNP | pO2,Blood |
| Cholesterol, LDL, Calculated | Osmolality, Urine |  |
| Creatine Kinase (CK) | Oxygen Saturation |  |
| Creatine Kinase, MB Isoenzyme | Oxygen |  |
| Creatinine, Urine | PTT |  |
| D-Dimer | Parathyroid Hormone |  |
| Ferritin | Protein |  |
| Fibrinogen, Functional | RBC, Ascites |  |

**Supplemental Table 2**: Feature selection fields for text data. Text items for feature selection. 47 important features were selected from the text data using the LASSO method.

| abuse | dialysis | mitral |
| --- | --- | --- |
| aicd | diastolic | normal |
| amiodarone | digoxin | obstructive |
| atrial | dilated | severe global |
| atrial fibrillation | dilated cardiomyopathy | severely depressed |
| back | diltiazem | shock |
| bleeding | hct | surgery |
| captopril | hypertrophic | syndrome |
| cardiac | hypokinesis | transplant |
| cardiomyopathy | incision | valve |
| carvedilol | insulin | ventricular |
| clonidine | ischemic |  |
| cocaine | lasix |  |
| congestive | left |  |
| coronary | mca |  |
| coronary artery | mild |  |
| coronary artery disease | mildly depressed |  |
| delayed | milrinone |  |


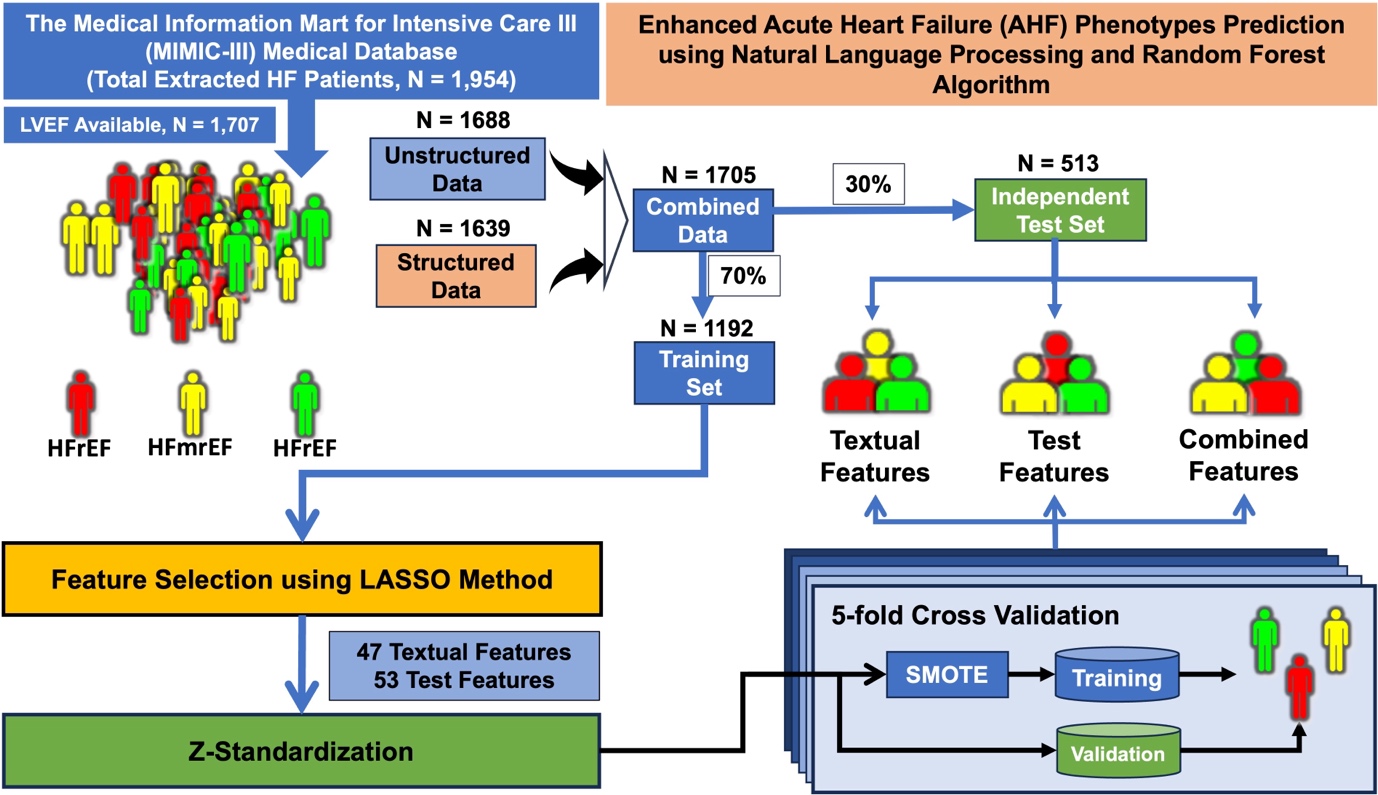


**Supplemental Figure 1:** Workflow for developing AHF subtype prediction models. The dataset was composed by merging structured and unstructured data corresponding to 1,639 and 1,688 patients, respectively. This integration yielded a cohort of 1705 unique HF patients. To prepare the dataset for model development, stratified random sampling was employed to partition the data into training (70%) and independent test (30%) sets. The process begins with feature selection, where unstructured textual data and biochemical test data from the training set are refined using the LASSO method. This step identifies 47 key textual features and 53 biochemical test features. Subsequently, we construct three variants of random forest models: one using only textual data, another using only biochemical data, and a third that combines both data types. We employed a five-fold cross-validation method for the training and validation of our models. Additionally, the SMOTE was used to balance the training data.

A.


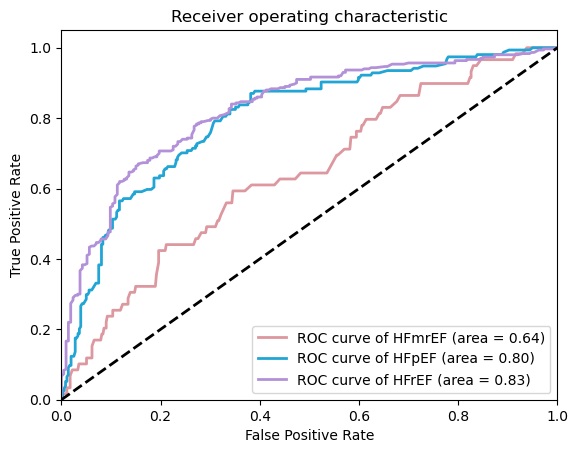

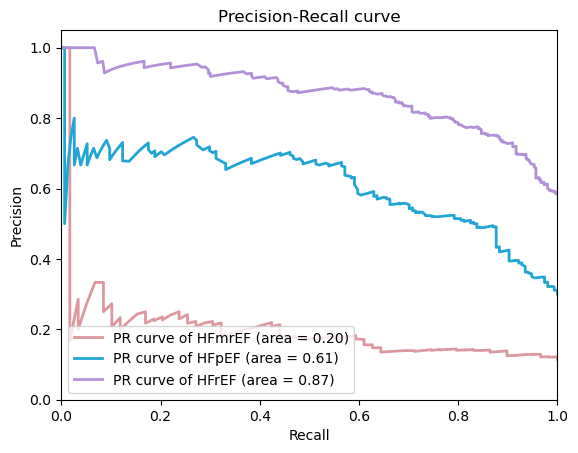


B.


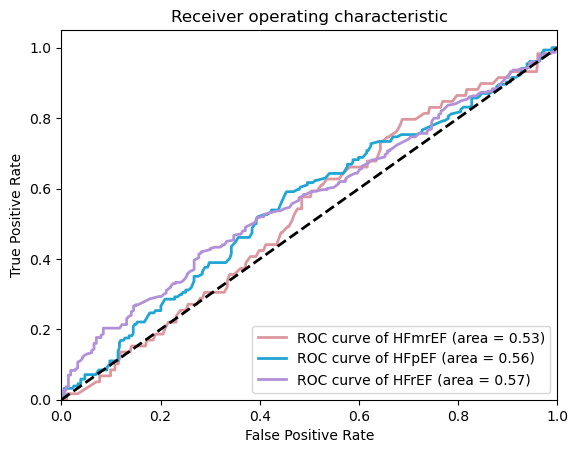

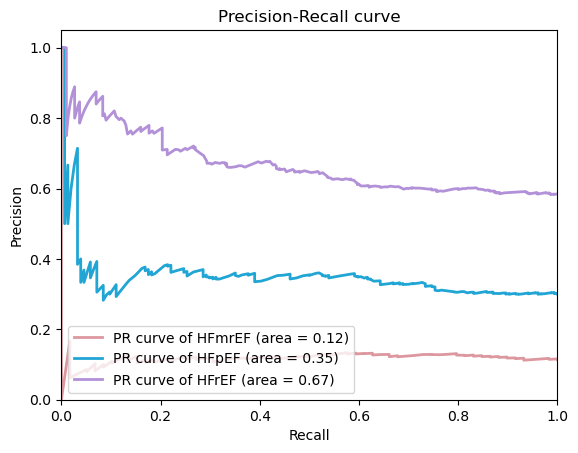


C.


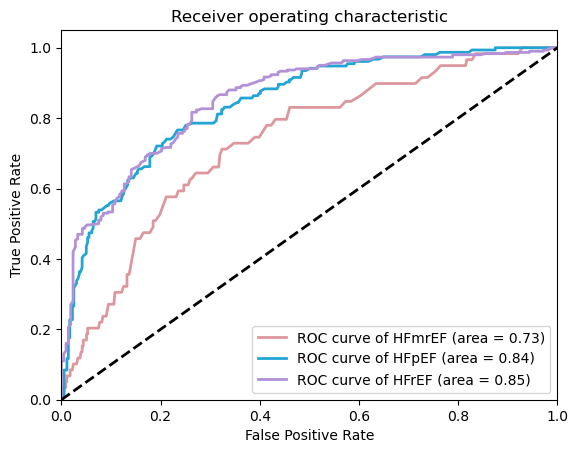

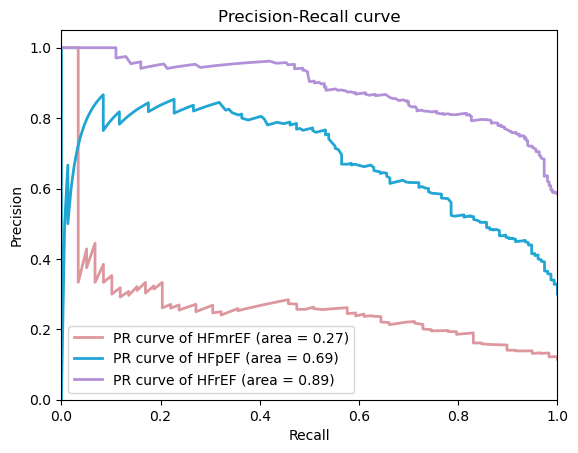


**Supplemental Figure 2:** Performance of random forests for HF phenotype classification with reduced features using different data configurations. (A) illustrates the effectiveness of random forest models using textual data with 23 features, (B) depicts models using laboratory test data with 26 features, and (C) shows models combining textual and laboratory test data with 50 features. Each subfigure includes ROC curves on the left and PRC curves on the right.

Appendix C:


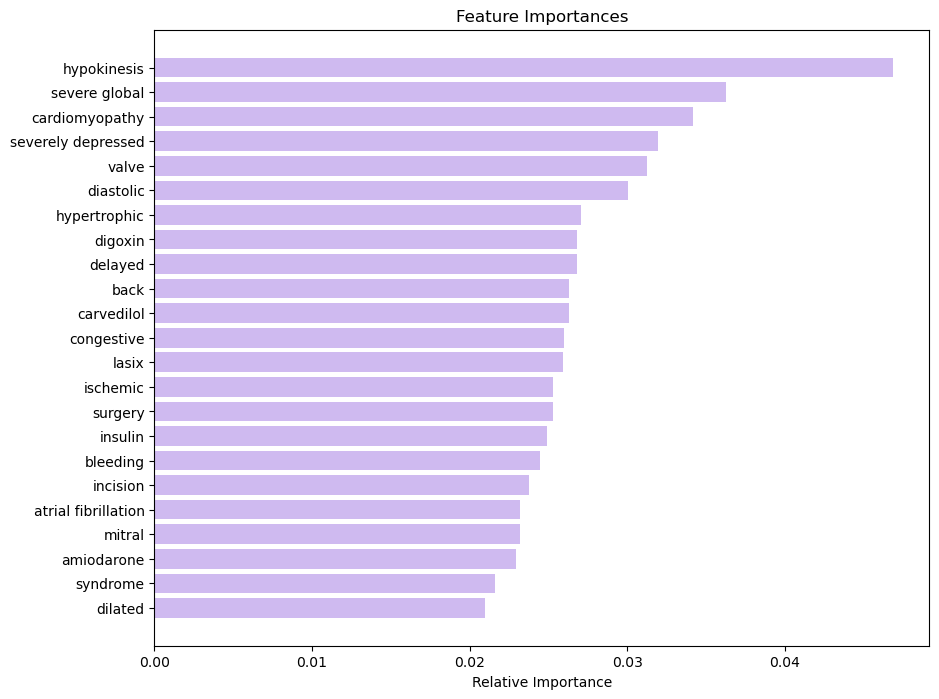


**Supplemental Figure 3A.** Feature importance of text data. The top 23 features of the original text data ranked by importance under the random forest model, sorted from top to bottom.


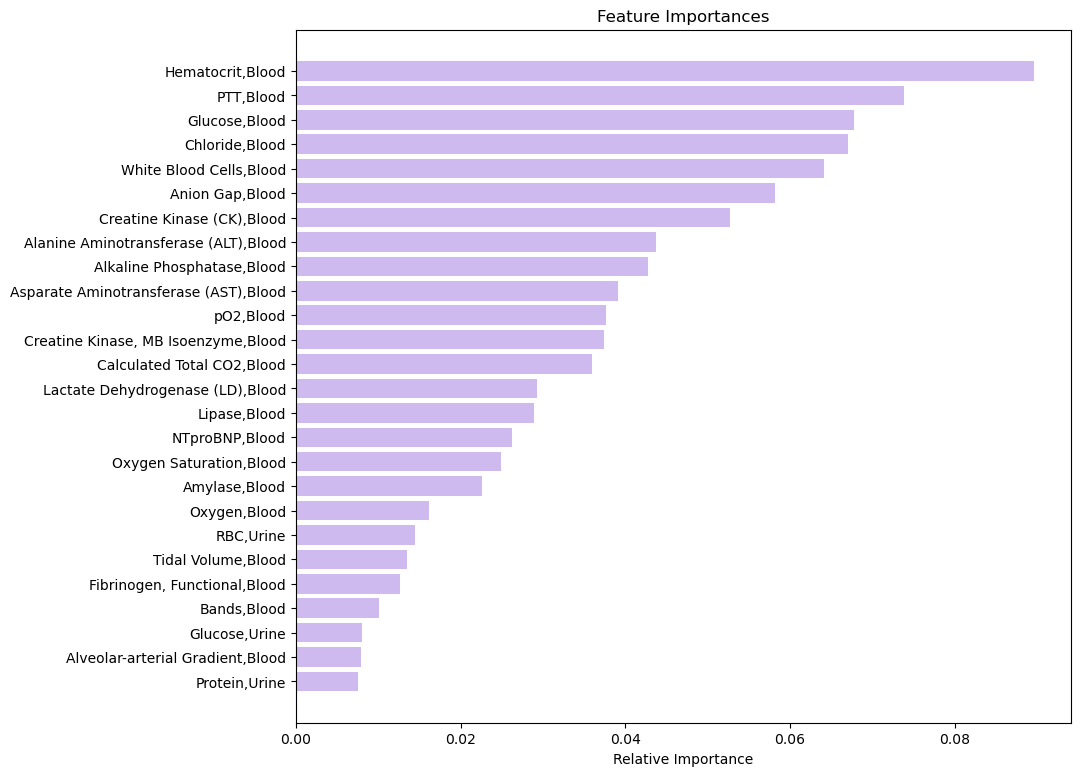


**Supplemental Figure 3B.** Feature importance of examination data. The top 26 features of the original examination data ranked by importance under the random forest model, sorted from top to bottom.


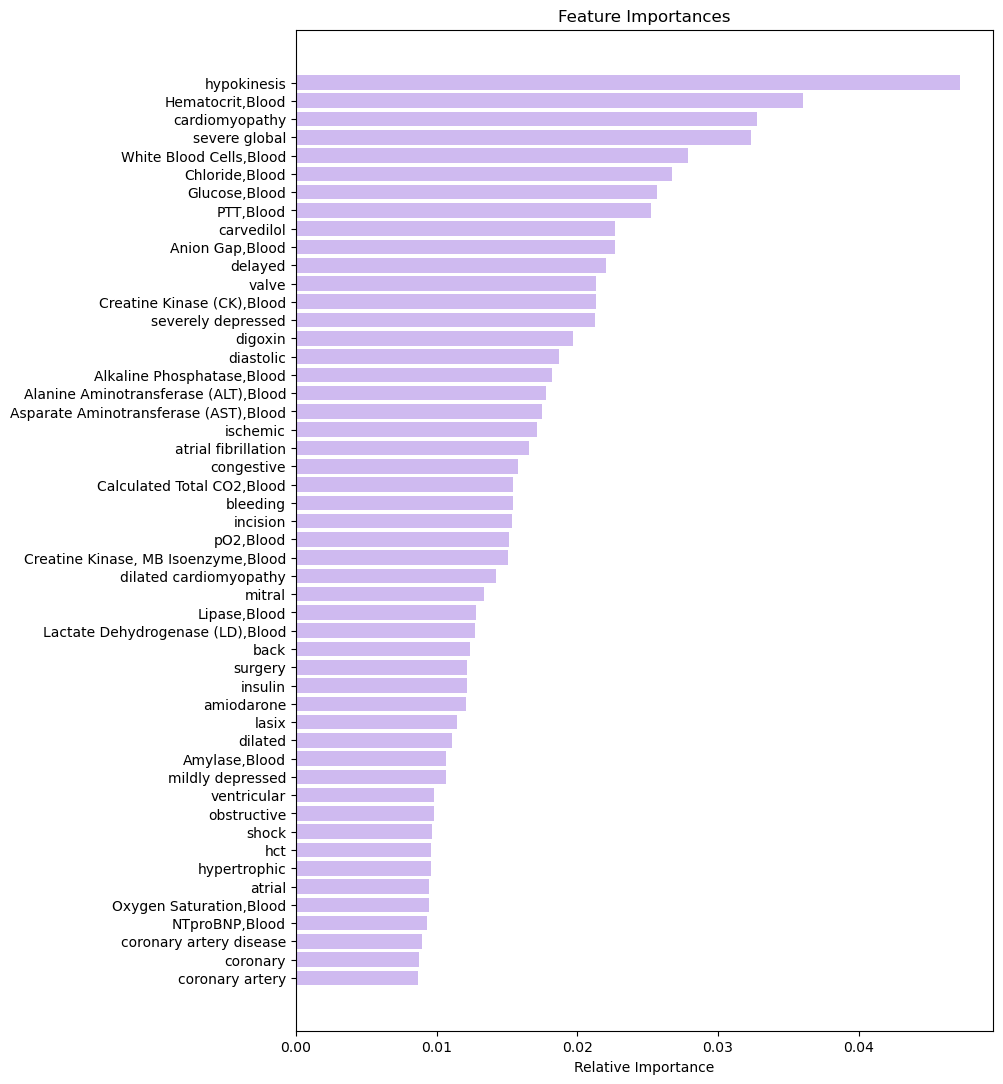


**Supplemental Figure 3C.** Feature importance of both data types. The top 50 features of both text and examination data ranked by importance under the random forest model, sorted from top to bottom.

Reference

1. Tibshirani, R., *Regression Shrinkage and Selection via the Lasso.* Journal of the Royal Statistical Society. Series B (Methodological), 1996. **58**(1): p. 267-288.
2. Breiman, L., *Random Forests.* Machine Learning, 2001. **45**(1): p. 5-32.
3. Velazquez EJ, Morrow DA, DeVore AD, et al. Angiotensin–neprilysin inhibition in acute decompensated heart failure*.* *N Engl J Med*. 2018;380:539-548.
